# Supplementary material for: Effect of Yeast Fermentation on the Physicochemical Properties and Bioactivities of Polysaccharides of Dendrobium officinale
Source: Foods. 2022 Dec 28;12(1):150. doi: 10.3390/foods12010150 (PMC9818654; doi:10.3390/foods12010150)
Supplement: Supplementary file 1 [file foods-12-00150-s001.zip › foods-1994868-supplementary.pdf]

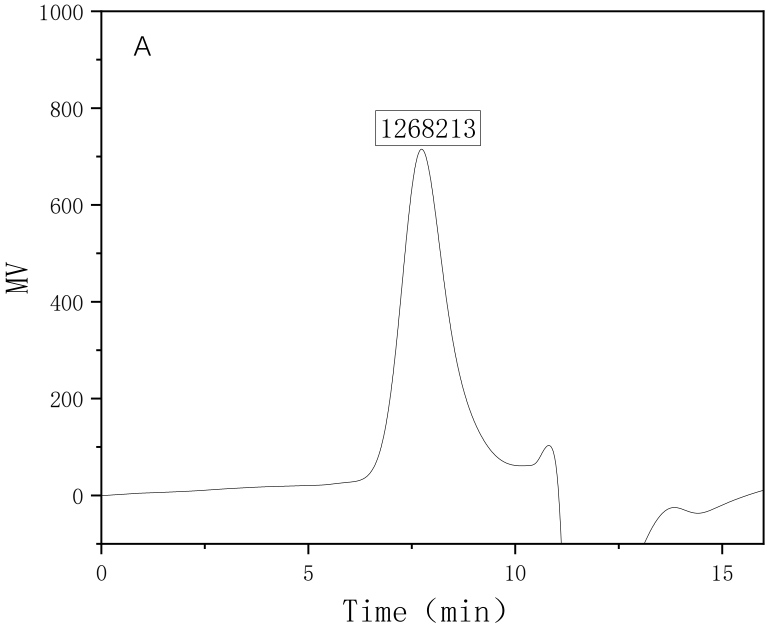
A
1268213
MV
1000
800
600
400
200
0
0
5
10
15
Time (min)

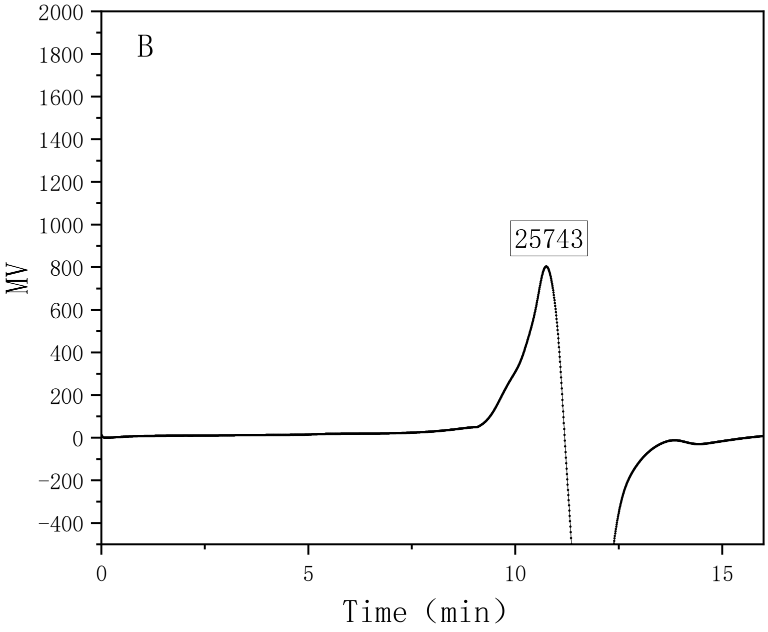
B
25743
MV
2000
1800
1600
1400
1200
1000
800
600
400
200
0
-200
-400
0
5
10
15
Time (min)

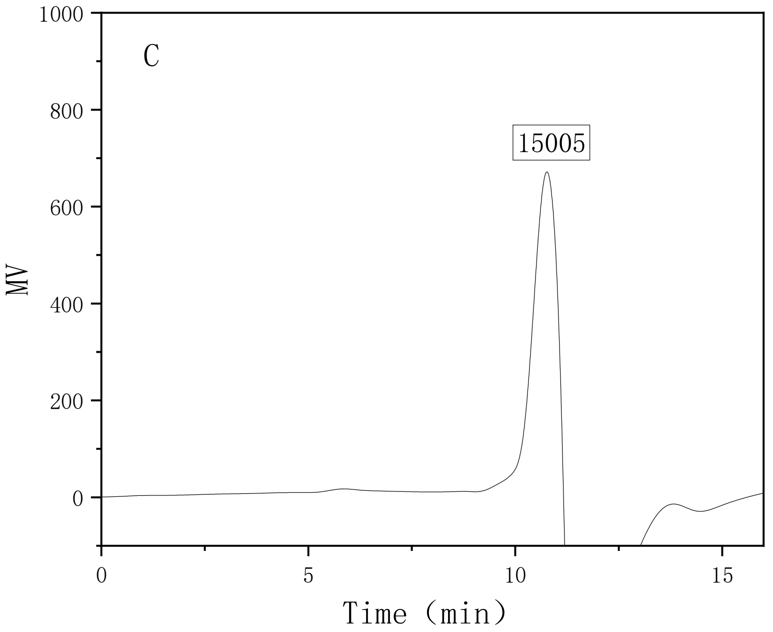

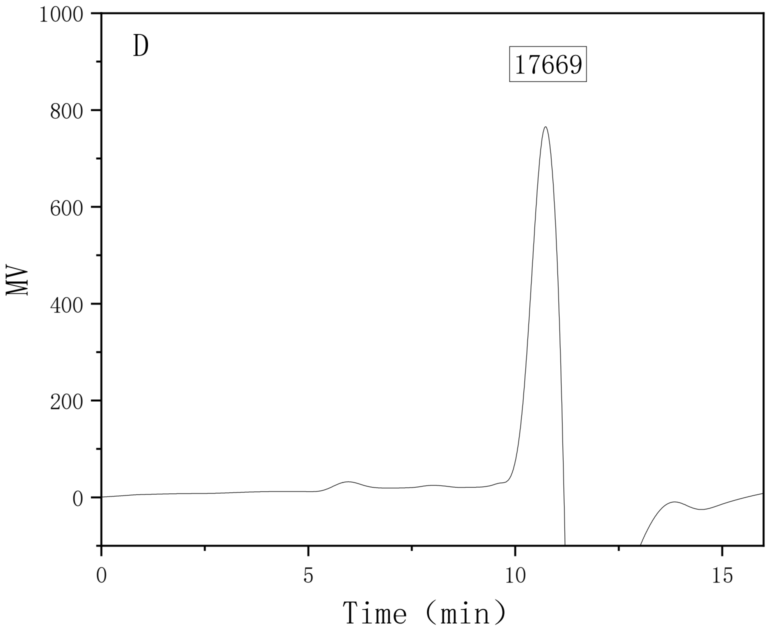

Figure S1. molecular weight distribution of DOP (**A**), DOSCP (**B**), DOWAP (**C**)and DOSWP (**D**)

Table S1. Polysaccharide molecular weight distribution.

Sample	Mn (KDa)	Mw (KDa)	Mp (KDa)	Mw/Mn
DOP(A)	637.09±43.51	25.74±0.21	1182.48±99.34	1.99
DOSCP(B)	16.45±0.73	15.01±0.53	12.79±0.93	1.56
DOWAP(C)	13.15±0.53	17.67±0.34	12.73±0.65	1.14
DOSWP(D)	14.43±0.48	1268.21±10.23	13.07±0.43	1.22
